# Pharmacogenomics at the post-pandemic: If not now, then when?

**DOI:** 10.3389/fphar.2022.1013527

**Published:** 2022-09-26

**Authors:** Zeina N. Al-Mahayri

**Affiliations:** Department of Genetics and Genomics, College of Medicine and Health Sciences, United Arab Emirates University, Al Ain, United Arab Emirates

**Keywords:** pharmacogenomics (PGx), COVID-19, precision medicine, cost-effective, understudied groups

The emergence of Coronavirus disease 2019 (COVID-19) has changed the world in an irrevocable direction. The changes have affected all vital sectors, including finance, education, communications, research, and, undoubtedly, the medical practice. Every field related to human health explored how to escalate its performance and updated its research priorities to respond to the situation imposed by the devastating pandemic. Pharmacogenomics (PGx) is not an exception. Multiple researchers in the field investigated the available PGx information related to medications used in COVID-19 management, and various reviews have appeared since late 2020 till today (Takahashi et al., 2020; Badary, 2021; Fricke-Galindo and Falfán-Valencia, 2021; Biswas et al., 2022; Franczyk et al., 2022). Most of these reviews concluded that the evidence of the pharmacogenomic interactions for drugs used in the new Coronavirus-induced severe respiratory syndrome (SARS-CoV2) infection management is not strong enough to warrant testing. Herein, there are common findings among these reviews that deserve highlighting. Moreover, extrapolating these findings into populations underrepresented in PGx research that were challenged by the pandemic, equally to all other populations, can reflect on future practices. The following insights explore the probability of the situation imposed by COVID-19 being an opportunity, rather than a threat, for largescale PGx adoption.

The earliest publication of SARS-CoV2 treatment PGx was a literature review by Takahashi and colleagues (Takahashi et al., 2020), which was available online in August 2020. In their work, the authors listed the available therapeutic options for SARS-CoV2 infection used in the US healthcare system and in interventional clinical trials registered on ClinicalTrials.gov at the time of publication. The authors reviewed the pharmacogenetic biomarkers found to be associated with these drugs. None of the studied drugs had approved PGx-guided dosing guidelines, and few had moderate to low significance PGx associations. The illustrated candidate response and toxicity biomarkers were retrieved from previous research in diseases other than COVID-19. The authors highlighted that no PGx data is available from SARS-CoV2 patients yet.

Nevertheless, Takahashi and colleagues used the association between abacavir, the anti-HIV agent, and *HLA*-B*57:01 to exemplify the success of pharmacogenomics implementation in infectious diseases. At the same time, they underscored the time between identifying HIV and recognizing this PGx association and its implementation success (Takahashi et al., 2020). Compared to HIV-induced acquired immunodeficiency, the COVID-19-induced illness burden on global public health is unmatched, and the urgent need for better control measures is substantially different.

On the other hand, and due to limited PGx data for most of the antiviral drugs re-purposed for COVID-19, some reviews illustrated the available sporadic reports about PGx associations from the literature (Badary, 2021; Fricke-Galindo and Falfán-Valencia, 2021; Franczyk et al., 2022). The listed studies’ outcomes were primarily inconclusive and limited by sample size or conflicting results. Nevertheless, demonstrating them collectively displays the current deficiency in PGx research on drugs used in infectious diseases compared to PGx research on non-communicable conditions medications.

One common outcome of the COVID-19-PGx reviews is listing the candidate PGx effectors on drugs used for SARS-CoV2. For example, SLCO1B1*4 was recorded as a candidate effector on lopinavir clearance. The variations in this allele frequency among populations were briefly highlighted, and its frequency was reported as 14% in Europeans, 6% in Africans, and 0.3% in East Asians (Takahashi et al., 2020). However, in some Middle Eastern populations, this allele’s prevalence reaches 20%–45% (Pasanen et al., 2008; Al-Mahayri et al., 2020). The high prevalence of a deficient *SLCO1B1* allele could be associated with increased clearance of lopinavir in these ethnicities. Although there has been a recommendation against using lopinavir in SARS-CoV2 in the early 2021 (COVID-19 Treat Guidelines, 2022), *SLCO1B1* is active in the transportation of other antivirals*,* like atazanavir.

Furthermore, in a more recent SARS-CoV2 drugs PGx review (Biswas et al., 2022), the authors went beyond the PGx interactions of first-line COVID-19 used therapeutics to consider all medications used in supportive therapy, drug-drug interactions, drug-herbal interactions, besides the genetic biomarkers of disease severity. In their review, Biswas and colleagues listed the most critical PGx-biomarkers that interact with drugs used in COVID-19 main therapeutic agents (i.e., antivirals) and supportive therapies (i.e., corticosteroids, antiplatelets, anticoagulants, non-steroidal anti-inflammatory agents, etc.). Accordingly, they suggested a panel of genes of interest that can be used to assess the impact of PGx interactions on COVID-19 and to establish COVID-19 precision medicine. These genes include *CYP3A4/5, CYP2B6, CYP2C8, CYP2C9, CYP2C19, CYP2D6, ABCB1,* and *SLCO1B1,* among others. These specific genes illustrate a wide range of inter-population and inter-ethnic variability, as reported in studies from Africa and Southeast Asia (da Rocha et al., 2021; Runcharoen et al., 2021). Table 1 lists some of these variants and demonstrates the difference in minor allele frequencies between Europeans, commonly represented in genetic data, and selected examples of rarely studied populations. Indeed, the lack of diversity in genomic data has an unforeseen effect on global health. For instance, although the African populations exhibit the most diverse genomes among human populations, the most minor data about COVID-19 host genomes came from Africa. Simultaneously, African populations suffer from the highest burden of infectious diseases (Zhang et al., 2022). However, whether the PGx variations in the actionable genes among populations can explain any differences in COVID-19 treatment outcomes was not evaluated, though not possible to exclude given the lack of such studies.

**TABLE 1:** Examples of the suggested gene-drug pairs related to the management of COVID-19 and the frequencies of their minor alleles in Europeans compared to selected rarely-studied populations.

Drug	Gene	Variant	PharmGKB association [level of evidence]^a^	European (non-Finish)	Arabs from the UAE	Southeast Asians from Indonesia	Southeast Asians from the Philippines	Black Africans from Zimbabwe
Atazanavir	*SLCO1B1*	rs4149056 (*5)	Genotype TT is associated with the dose of atazanavir in people with HIV Infections [NA]	0.1589	0.1869	0.1	0.083	0.005
	*UGT1A1*	rs4148323 (*6)	Patients carrying the *6 allele in combination with another decreased function allele may have increased likelihood of hyperbilirubinemia when treated with atazanavir (in most studies boosted with low dose of ritonavir) as compared to patients with two normal function alleles. [1A]	0.002	0.005	0.03	0.031	NA
Azithromycin	*ABCB1*	rs1045642	Genotype AA is associated with decreased concentrations of azithromycin in healthy individuals as compared to genotype GG [NA]	0.4663	0.5969	0.614	0.714	0.09
Clopidogrel	*CYP2C19*	rs4244285 (*2)	Patients carrying the no function allele in combination with a no, decreased, normal, or increased function allele who are treated with clopidogrel may have an increased risk for adverse cardiac and cerebrovascular events as compared to patients with two normal function alleles [1A]	0.1468	0.1515	0.241	0.077	0.17
Dexamethasone	*UGT1A1*	rs4148323 (*6)	—	0.002	0.005	0.03	0.031	NA
	*ABCB1*	rs1045642	—	0.4663	0.5969	0.614	0.714	0.09
Efavirenz	*CYP2B6*	rs3745274	Patients with the GT or TT genotypes may have an increased risk of efavirenz-induced side effects, including sleep- and central nervous system-related side effects, as compared to patients with the GG genotype [1A]	0.2402	0.31	0.392	0.327	0.35
Ivermectin	*ABCB1*	rs1045642	-	0.4663	0.5969	0.614	0.714	0.09
Lopinavir	*ABCC2*	rs8187710	Patients with CT or TT genotype may have an increased risk of lopinavir toxicity [3]	0.05	0.1414	0.0055	—	NA
Losartan	*CYP2C9*	rs1799853 (*2)	Patients carrying the decreased function allele may have decreased metabolism of losartan as compared to patients with the normal function allele [3]	0.1262	0.11	0.0009	—	0.01
	*ABCB1*	rs1045642	Patients with the GG genotype may have poorer response to losartan in people with hypertension as compared to patients with the AA or AG genotype [3]	0.4663	0.5969	0.614	0.714	0.09
Ribavirin	*SLC28A2*	rs11854484	Patients with the TT genotype (and hepatitis C) may have an increased risk for anemia when treated with protease inhibitors plus ribavirin and peginterferon, as compared to patients with the CC or CT genotype [3]	0.6216	0.4286	0.222	0.18	NA
Warfarin	*CYP2C9*	rs1799853 (*2)	Patients with decreased function allele with another normal, decreased, or no function allele may have increased risk of over-anticoagulation when treated with warfarin as compared to patients with two normal function alleles	0.1262	0.11	0.0009	—	0.01
References			PharmGKB (2022)	gnomAD (2022)	Al-Mahayri et al. (2020)	Runcharoen et al. (2021)	Runcharoen et al. (2021)	Muyambo et al. (2022)

NA; not available, PharmGKB; pharmacogenomics knowledge base.

a Level of evidence: according to the PharmGKB grading system.

According to the mentioned COVID-19 PGx reviews, a significant limitation hindering PGx data use for COVID-19 patients is the unavailability of the patient’s PGx profile when admitted for acute illness. Pre-emptive PGx testing would be one solution for this limitation, while providing a reactive point-of-care PGx testing is another. Stevenson and colleagues have shown that in a cohort of patients hospitalized for COVID-19, nine in 10 patients had at least one order of a drug with a PGx recommendation. Moreover, through a simulation analysis, the authors found that 17 treatment modifications per 100 patients would be feasible if pre-emptive PGx data were available in the EHRs of these patients at the time of admission. The authors also emphasized that the multigene-PGx results are projected to positively impact patients’ healthcare years after hospital discharge (Stevenson et al., 2021).

In a similar attempt to explore the PGx-data potential impact on COVID-19 outcomes, Sahana and coworkers analyzed the PGx variants from the “IndiGen” dataset, the dataset of 1000 + Indian whole genome sequences. The authors concluded that the population-specific PGx variation landscape, if utilized, could have contributed to designing population-specific clinical trials and expediting decision-making throughout the pandemic (Sahana et al., 2021).

Another critical point to consider, specifically during a pandemic when few therapeutic options are available, is that any PGx testing is limited by proving its cost-effectiveness (Takahashi et al., 2020). However, if preemptive PGx testing had been adopted and integrated into the health records, PGx data would have proved its cost-effectiveness, and such limitations would have been eliminated. It has been repeatedly shown that almost 96%–99% of healthy individuals carry an actionable PGx variant if tested through a multigene testing (Chanfreau-Coffinier et al., 2019; Tasa et al., 2019).

Nevertheless, even if the previous controversial points were resolved and the PGx data is available at the point of admission, it cannot be utilized unless clinical guidelines are available at the hands of the clinician. This fact brings us back to the difficulty in generating such guidelines without extensive prospective studies, which should prove cost-effectiveness besides positive or equivalent clinical outcomes. There is a severe need to break this vicious circle before a new pandemic strikes. Utilizing novel machine learning approaches (Gaziano et al., 2021) and integrating epidemiological methodologies like Mendelian randomization (Khasawneh et al., 2022) can offer much-needed supporting evidence. Machine learning promises to fill the gap between translational research and clinical practice, which can empower healthcare systems through analyzing real-life data and building prediction models (Santus et al., 2021). Nevertheless, suppose such unconventional approaches fail to gain the practitioners’ and regulators’ confidence or fail to harness ethical concerns. In that case, PGx implementation might be stuck in the same vicious circle for a long time.

Interestingly, COVID-19 patients’ DNA biorepositories have already been established (COVID-19 hg, 2022). While these were collected to fuel COVID-19 research, extracting PGx data and returning it to patients’ HER should be considered whenever the collected consents and the governing regulations allow such a practice (Stevenson et al., 2021). Indeed, utilizing clinical and genomic data gathered during the pandemic can unleash opportunities for precision medicine. For example, the retrospective analysis of clinical and genomic data of 4,125 patients hospitalized for COVID-19 showed that intermediate or poor CYP2C19 metabolizers treated with remdesivir experienced higher alanine aminotransferase (ALT) elevations compared to normal, rapid, or ultrarapid metabolizers. Due to its emergency approval for COVID-19, there was limited data about remdesivir safety or pharmacokinetics before this report (Tuteja et al., 2022). Accordingly, this recent finding about *CYP2C19*-remdesivir interaction exemplifies the potential gains of the availability of PGx information in patients’ health records.

Herein, the minimal COVID-19 PGx available data could be seen as an unprecedented motivation to accelerate the PGx implementation process. With the decrease in genetic testing costs, the increase in the number of available techniques for genotyping, and the evolution in data science that allows optimal data storage and making sense of genetic information, there is an immense need to consider PGx testing in the population scale and maintain this information in medical health records. Having this information at the point of admission due to an acute illness or in the mid of a pandemic is, undoubtedly, a rich source for evaluating the practice of precision medicine in real life. While developed countries with more robust healthcare systems can be considered the ultimate candidates for taking the lead in PGx implementation, developing countries and those with emerging economies inhabited by poorly studied communities should explore investing in population-PGx as a plausible approach to achieving cost-effective precision health care.

To conclude, before the next pandemic actively hits, there is a need to seriously consider preemptive PGx testing implementation in different populations. Postponing such initiatives due to doubts about cost-effectiveness or performance feasibility threatens time, money, and probably the lives of people who could have been spared from unnecessary treatments or adverse drug events.
